# NMDA Reduces Tau Phosphorylation in Rat Hippocampal Slices by Targeting NR2A Receptors, GSK3**β**, and PKC Activities

**DOI:** 10.1155/2013/261593

**Published:** 2013-11-20

**Authors:** Audrée De Montigny, Ismaël Elhiri, Julie Allyson, Michel Cyr, Guy Massicotte

**Affiliations:** Groupe de Recherche en Neuroscience, Département de Biologie Médicale, Université du Québec à Trois-Rivières, Trois-Rivières, QC, Canada G9A 5H7

## Abstract

The molecular mechanisms that regulate Tau phosphorylation are complex and currently incompletely understood. In the present study, pharmacological inhibitors were deployed to investigate potential processes by which the N-methyl-D-aspartate (NMDA) subtype of glutamate receptors modulates Tau phosphorylation in rat hippocampal slices. Our results demonstrated that Tau phosphorylation at Ser199-202 residues was decreased in NMDA-treated hippocampal slices, an effect that was not reproduced at Ser262 and Ser404 epitopes. NMDA-induced reduction of Tau phosphorylation at Ser199-202 was further promoted when NR2A-containing receptors were pharmacologically isolated and were completely abrogated by the NR2A receptor antagonist NVP-AAM077. Compared with nontreated slices, we observed that NMDA receptor activation was reflected in high Ser9 and low Tyr216 phosphorylation of glycogen synthase kinase-3 beta (GSK3**β**), suggesting that NMDA receptor activation might diminish Tau phosphorylation via a pathway involving GSK3**β** inhibition. Accordingly, we found that GSK3**β** inactivation by a protein kinase C- (PKC-) dependent mechanism is involved in the NMDA-induced reduction of Tau phosphorylation at Ser199-202 epitopes. Taken together, these data indicate that NR2A receptor activation may be important in limiting Tau phosphorylation by a PKC/GSK3**β** pathway and strengthen the idea that these receptors might act as an important molecular device counteracting neuronal cell death mechanisms in various pathological conditions.

## 1. Introduction

Over the years, a growing number of reports have revealed that, in contrast to the destructive effects of excessive N-methyl-D-aspartate (NMDA) receptor activity, synaptic NMDA receptor stimulation under physiological conditions could result in the activation of prosurvival mechanisms in neurons [1–5]. For instance, it appears that tonic activation of NMDA receptors in hippocampal neurons is required for maintaining synaptic stability, through a mechanism involving modulation of dendritic protein synthesis [6]. In fact, it has been proposed that the tonic activity of NMDA receptors is a crucial mechanism regulating calcium mobilization in neurons, as NMDA receptor deprivation rapidly increases the synaptic expression of surface GluR1 subunits and the incorporation of toxic Ca^2+^-permeable *α*-amino-3-hydroxy-5-methyl-4-isoxazolepropionate (AMPA) receptors at glutamatergic synapses [7, 8].

Fiumelli et al. [9] demonstrated that suppression of NMDA receptor activity by global antagonists (MK801 or AP5) can interfere with both phosphorylation and solubility of neurofilament subunit M in isolated cortical neurons. In this particular case, neurite outgrowth appears to be promoted by the inactivation of NMDA receptors, suggesting that the basal levels of NMDA receptor activity are crucial for regulating cytoskeleton stability and growth processes. Other data suggest that tonic NMDA receptor activity, in cerebellar granule cells and hippocampal neurons, regulates microtubule-associated protein 2 (MAP2) phosphorylation and neurite growth [10–12], while some authors have shown that activation of NMDA receptors in physiological conditions is capable of influencing Tau phosphorylation in the hippocampal area [9–11, 13]. In healthy neurons, Tau proteins are well-known for their involvement in the outgrowth of neural processes, axonal transport, development of neuronal polarity, and maintenance of normal neuron morphology [14–16], whereas many neurodegenerative diseases are characterized by Tau hyperphosphorylation, Tau (mis)localisation in neurons, and, consequently, the development of neurofibrillary tangles [17].

Although the detailed molecular mechanisms by which NMDA receptors can regulate both physiological and pathophysiological processes remain to be elucidated, it has been proposed that NMDA receptor function may be highly dependent on the composition of their subunits, which are heteromeric assemblies of at least one NR1 subunit and various NR2 (A–D) subunits [18–21]. In the hippocampus, extensive evidence indicates that, in the mature stage, pyramidal cells mainly express NMDA receptors containing NR1/NR2A and NR1/NR2B subunits [22, 23]. From a functional perspective, it has been argued by many that physiological NR1/NR2A subunit activation could favour the participation of prosurvival mechanisms, whereas excessive NR1/NR2B subunit stimulation could lead to neuronal cell death by the involvement of various damaging signaling pathways [5, 24, 25].

Using different pharmacological agents, we reported previously that tonic stimulation of NR2A-containing NMDA receptors in hippocampal slices might be a crucial component limiting Tau hyperphosphorylation [26]. To gain further insight into this effect, we investigated how NMDA treatments modify different Tau isoforms at various phosphorylation sites, specifically those recognized by antibodies raised against Ser199-202, Ser262, and Ser404 Tau epitopes. The contribution of different signaling pathways regulating Tau phosphorylation through GSK3*β* activity was also examined.

## 2. Materials and Methods

### 2.1. Ethics Approval

Animal care procedures were reviewed by the Institutional Animal Care Committee of the Université du Québec à Trois-Rivières and determined to be in compliance with guidelines of the Canadian Council on Animal Care.

### 2.2. Animals and Pharmacological Agents

Male Sprague-Dawley rats (4-5 weeks of age), purchased from Charles River Laboratories (Montréal, QC, Canada), were housed for 1 week in a temperature-controlled room, prior to any experiments, with free access to laboratory chow and water. The selective NR2A antagonist NVP-AAM077 (NVP) was a gift from Dr. Yves Auberson (Novartis Pharma AG, Basel, Switzerland). The NR2B receptor antagonist RO25-6981 and the Akt/PKB (protein kinase B) inhibitor 10-[4′-(N,N-Diethylamino) butyl]-2-chlorophenoxazine hydrochloride (10-DEBC) were obtained from Tocris Bioscience (Ellisville, MO, USA), while the membrane-impermeable calcium  chelator  1,2-bis(o-aminophenoxy)ethane-N,N,N′,N′-tetraacetic acid (BAPTA) was procured from BioMol (Plymouth, PA, USA). Inhibitors of protein kinase C (PKC; Chelerythrine chloride), phosphoinositide 3-kinase (PI3K; LY294002), cyclin-dependent kinase 5 (cdk5; Roscovitine) as well as protease and phosphatase inhibitor cocktails were acquired from Calbiochem (San Diego, CA, USA).

### 2.3. Antibodies

Most antibodies reacting with Tau proteins were purchased from Abcam (Cambridge, MA, USA). The mouse polyclonal antibody Tau-5 (dilution 1 : 500) served to estimate total Tau protein levels in hippocampal extracts, along with rabbit polyclonal antibodies recognizing Tau phosphorylated at Ser199-202 (pSer199-202; dilution 1 : 1,000), Ser262 (pSer262; dilution 1 : 1,000), and Ser404 (pSer404; dilution 1 : 750). Total GSK3*β* (dilution 1 *μ*g/mL), GSK3*β* Ser9 (pSer9; dilution 1 *μ*g/mL), GSK3*β* Tyr216 (pTyr216; dilution 1 : 1,000), and *β*-actin antibody were also purchased from AbCam. Goat anti-rabbit or goat anti-mouse peroxidase-conjugated antibodies (dilution 1 : 5,000) and SuperSignal chemiluminescent substrate kits were from Pierce Chemical Co. (Rockford, IL, USA).

### 2.4. Hippocampal Slices and Tissue Samples

Sprague-Dawley rats were anesthetized by isoflurane inhalation (Baxter Corp., Toronto, ON, Canada) and decapitated. Their brains were quickly removed and placed in cold cutting buffer containing 126 mM NaCl, 3.5 mM KCl, 1.2 mM NaH_2_PO_4_, 2.3 mM MgCl_2_, 1 mM CaCl_2_, 25 mM NaHCO_3_, and 11 mM glucose, saturated with 95% O_2_/5% CO_2_ (pH 7.4). Coronal brain sections of 350 *μ*m, containing the hippocampus, were sliced in a Vibratome Series 1000 tissue sectioning system (Technical Products International, Inc., St. Louis, MO, USA). Sections were then transferred to artificial cerebrospinal fluid (ACSF) containing 126 mM NaCl, 3.5 mM KCl, 1.2 mM NaH_2_PO_4_, 1.3 mM MgCl_2_, 2 mM CaCl_2_, 25 mM NaHCO_3_, and 11 mM glucose, bubbled continuously with 95% O_2_/5% CO_2_ at 32°C. The brain sections were preincubated for 60 min before pharmacological treatment. After pharmacological treatment, hippocampal slices were dissected from the brain sections and homogenized in ice-cold RIPA lysis buffer containing 50 mM Tris-HCl, 150 mM NaCl, 1% Triton X-100, 0.25% sodium deoxycholate, and 1 mM EDTA supplemented with protease and phosphatase inhibitor cocktails.

### 2.5. Western Blotting

Protein levels extracted from rat hippocampus sections were measured by Bradford assay (Bio-Rad, Hercules, CA, USA). Protein lysates (40 *μ*g) were electrophoresed on 10% sodium dodecyl sulfate polyacrylamide gel (SDS-PAGE). Separated proteins were transferred onto nitrocellulose membranes, and nonspecific binding sites were blocked by incubation for 1 hour at room temperature in phosphate-buffered saline (PBS), pH 7.4, containing 5% bovine serum albumin (BSA fraction V) purchased from Fisher Scientific (Pittsburgh, PA, USA). Then, selected primary antibodies were incubated overnight at 4°C. After several washes with 0.1% Tween 20, the blots were incubated for 1 hour at room temperature in specific secondary horseradish peroxidase (HRP)-conjugated antibody solution. Both primary and secondary antibodies were diluted in Tris buffered saline ((TBS)/0.1% Tween 20/1% BSA). Immunoreactivity was visualized by chemiluminescence reactions and densitometric scanning with Vision Work LS software (UVP Bioimaging, Upland, CA, USA), and the intensity of the bands was quantified by ImageJ (W.S. Rasband, National Institutes of Health, Bethesda, MD, USA). The densitometry data were expressed as relative optical density.

### 2.6. Statistical Analysis

The results are expressed as mean ± S.E.M. Statistical significance of the changes was determined by Graph Prism version 5.0 (Graph Pad Software, San Diego, CA, USA). *P* < 0.05 values were considered as statistically significant.

## 3. Results

### 3.1. Tau Phosphorylation at Ser199-202 is Reduced by NMDA Treatment: Role of NR2A-Containing Receptors

**Figure 1:**
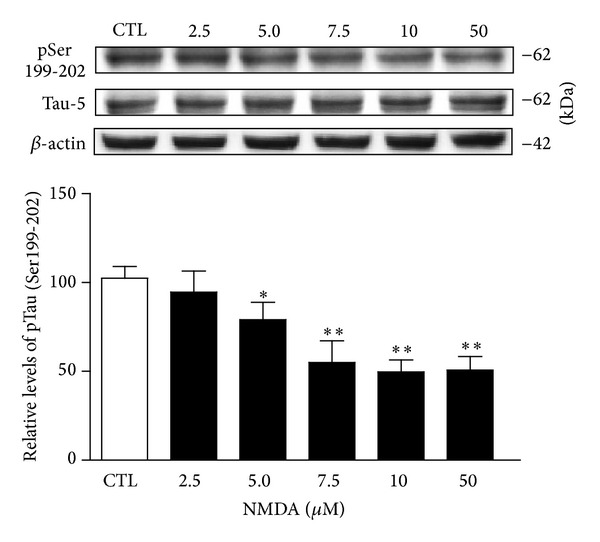
Tau phosphorylation at Ser199-202 sites is reduced after NMDA treatment. Phosphorylation and protein levels were estimated by Western blotting of cell extracts (40 *μ*g proteins) obtained from hippocampal slices treated with increasing concentrations of NMDA for 1 hour. Phosphorylated Tau levels at Ser199-202, expressed relative to total Tau (Tau-5) levels, were measured in slices treated with or without NMDA. The data were expressed as percentages of control values and are means ± S.E.M. of 3 measurements per cell extract obtained from 8 different rats. For statistical analysis, one-way ANOVA was followed by Newman-Keuls *post hoc *test. *P < 0.05, **P < 0.01, NMDA-treated versus control.

**Figure 2:**
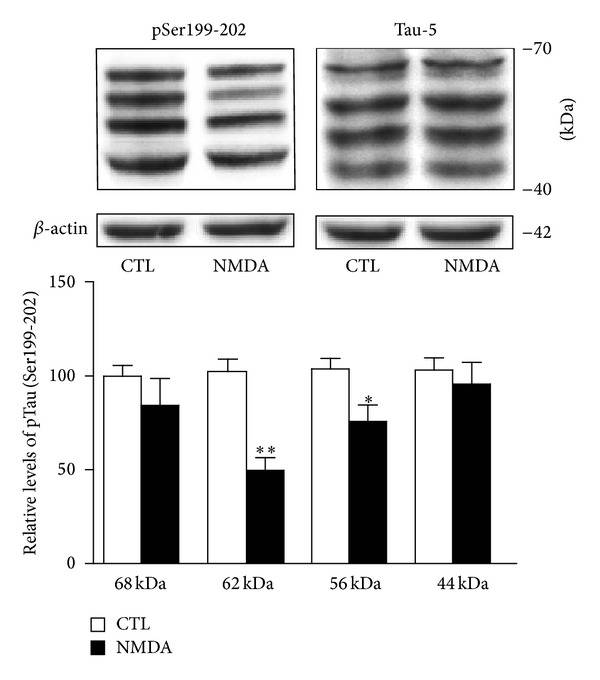
NMDA-induced changes in Tau phosphorylation is isoform-specific. Phosphorylated Tau levels at Ser199-202 were estimated by Western blotting of cell extracts obtained from acute hippocampal slices treated with or without 10 *μ*M NMDA for 1 hour. Four Tau isoforms were detected in blots developed with antibody directed against Ser199-202. Expressed relative to total Tau (i.e., Tau-5) levels, the data indicate that NMDA-induced reduction of Tau phosphorylation was striking for the 62-kDa isoform and, at a lesser degree, for the 56-kDa isoform. The data are expressed as percentages of control values and are means ± S.E.M. of 3 measurements per cell extract obtained from 8 different rats. Since each isoform was investigated independently, statistical significance was determined by unpaired *t*-test. *P < 0.05, **P < 0.01, NMDA-treated versus respective control.

To further explore the molecular mechanisms by which NMDA receptors might influence Tau phosphorylation, we assessed hippocampal slices kept metabolically active in oxygenated ACSF as model system. Hippocampal slices from rats were first preincubated for 1 hour with increasing NMDA concentrations ranging from 2.5 to 50 *μ*M, and Tau phosphorylation was then processed according to Western blotting procedures. In initial experiments, we observed that the Tau isoform, estimated to be 62 kDa, became progressively less phosphorylated at Ser199-202 residues when exposed to increasing NMDA concentrations. The threshold concentration for reducing Tau phosphorylation at Ser199-202 was in the order of 5 *μ*M with a maximal effect obtained at 10–50 *μ*M (Figure 1). Interestingly, a consistent feature in our experiments was the preferential modulation of Tau isoforms by NMDA, as depicted in Figure 2. NMDA treatments, in obvious contrast to 62- and 56-kDa Tau isoforms, produced no reliable changes in phosphorylation at Ser199-202 residues for the other two isoforms detected and estimated, with the help of molecular weight standards, to be around 44 and 68 kDa.

**Figure 3:**
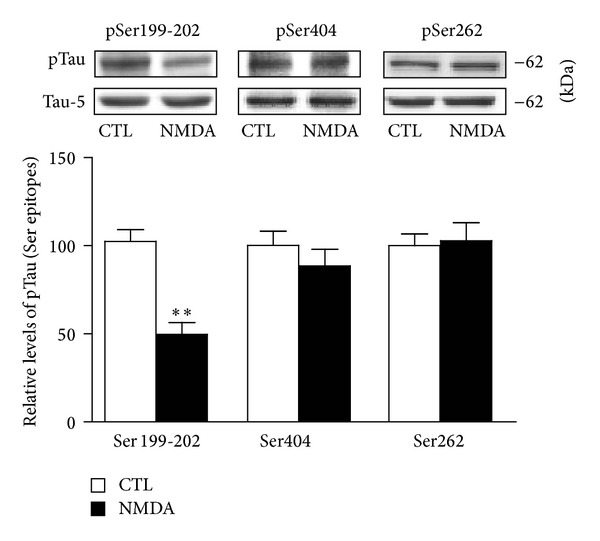
Tau phosphorylation at Ser262 and Ser404 residues is not altered by NMDA. Phosphorylation and protein levels were estimated by Western blotting of cell extracts (40 *μ*g proteins) obtained from hippocampal slices treated with 10 *μ*M NMDA for 1 hour. Phosphorylated Tau levels, expressed relative to total Tau (i.e., Tau-5) levels, were measured with antibodies raised against Tau phosphorylated at Ser199-202, Ser262, and Ser404. The data are expressed as percentages of control values and are means ± S.E.M. of 3 measurements per cell extract obtained from 6 different rats. Since these experiments were performed independently, we determined statistical significance by unpaired *t*-test. **P < 0.01, NMDA-treated versus control.

Tau has been found to possess more than 84 different phosphorylation sites [29–31]. Consequently, we tested whether NMDA treatment also affects other Tau epitopes.  Figure 3 shows that preincubation of hippocampal slices with 10 *μ*M NMDA for 1 hour failed to elicit changes in phosphorylation at Ser262 residues, a phosphorylation site positioned in the microtubule-binding domain of Tau proteins. Similarly, Western blotting experiments indicated that phosphorylation of an epitope located in the C-terminal domain of Tau, Ser 404, was not reduced after NMDA receptor activation (Figure 3).

**Figure 4:**
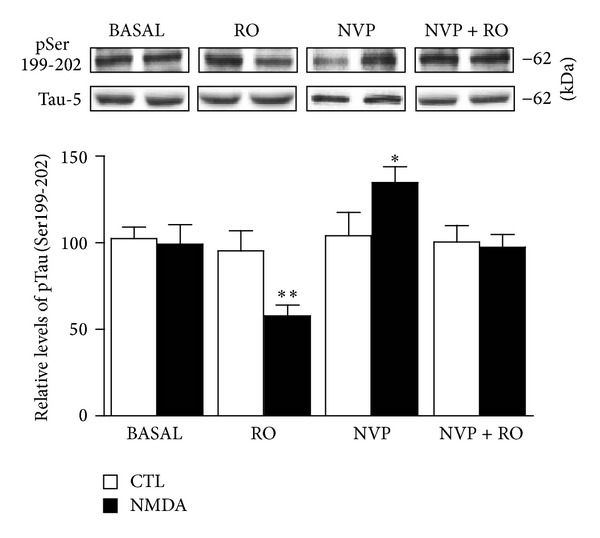
NMDA-induced changes in Tau phosphorylation are NR2A receptor-dependent. Phosphorylated Tau levels at Ser199-202 were estimated by Western blotting of cell extracts obtained from hippocampal slices. In basal conditions, slices were exposed to an NMDA concentration (1 *μ*M) which had no effect on Tau phosphorylation. In parallel experiments, NMDA-treated slices were preincubated with the NR2B receptor antagonist RO25-6981 (1 *μ*M), the NR2A receptor antagonist NVP-AAM077 (50 nM), or with a mixture of NR2B and NR2A (RO25-6981; 1 *μ*M and NVP-AAM077; 50 nM) receptor antagonists. Representative Western blots and quantitative data on each condition revealed that NMDA-induced reduction of Tau phosphorylation was exacerbated after blockade of NR2B receptors. On the contrary, Tau becomes hyperphosphorylated after blockade of NR2A receptors with NVP-AAM077 (50 nM). The data, expressed relative to total Tau (i.e., Tau-5) levels, are means ± S.E.M. of 3 measurements per cell extract obtained from 8 different rats. For statistical analysis, one-way ANOVA was followed by Newman-Keuls *post hoc* test. *P < 0.05, **P < 0.01, drug-treated versus control.

From a pharmacological perspective, it has been proposed that NR1/NR2A receptor activation could favour the action of prosurvival mechanisms as well as biochemical processes limiting Tau phosphorylation. The possibility that stimulation of NR2A-containing NMDA receptors is responsible for downregulating Tau phosphorylation was then considered.  Figure 4 illustrates that the ability of NMDA to reduce Tau phosphorylation was further enhanced in slices preexposed to the NR2B antagonist. In particular, pretreatment with RO25-6981 resulted in significant declines of phosphorylation levels of Tau at Ser199-202 with a low NMDA concentration (1 *μ*M), which normally did not alter phosphorylation levels of Tau at these epitopes. In contrast, we observed that blockade of NR2A receptors with NVP-AAM077 induces Tau phosphorylation at Ser199-202 epitopes. These data are indeed consistent with the notion that, when pharmacologically isolated, NR2A- and NR2B-containing receptors may exert opposite effects on Tau phosphorylation [26].

### 3.2. NMDA-Induced Regulation of Tau Phosphorylation: Role of Calcium and GSK3*β*

**Figure 5:**
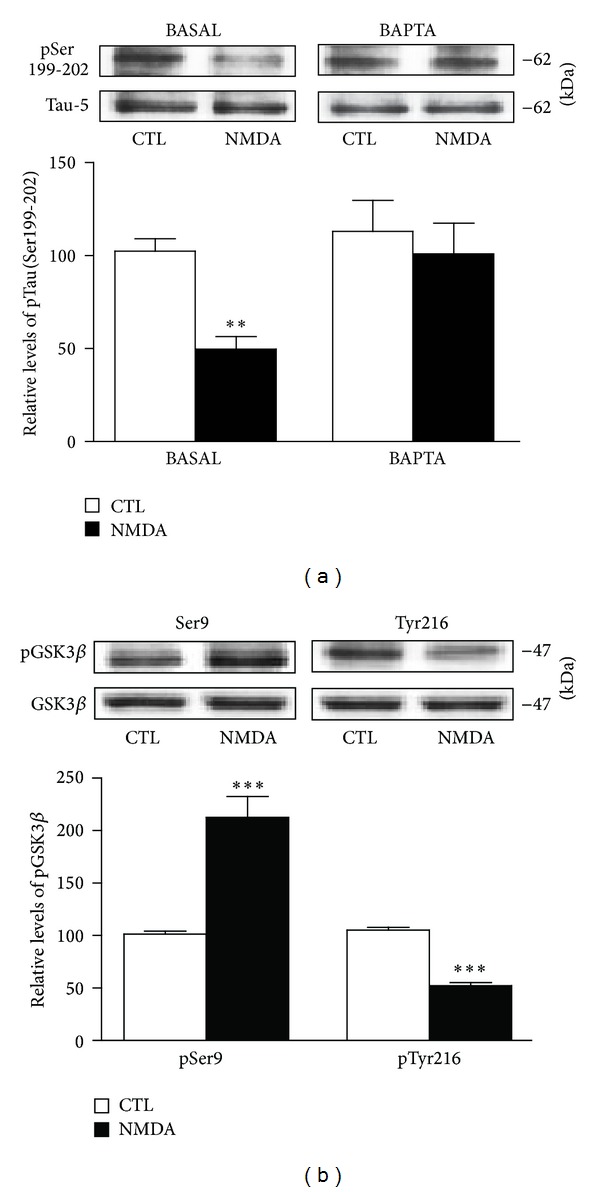
NMDA-induced changes in Tau phosphorylation are mediated by calcium and GSK3*β*. (a) Phosphorylated Tau levels at Ser199-202 were estimated by Western blotting of cell extracts obtained from hippocampal slices treated with 10 *μ*M NMDA for 1 hour alone or in combination with 10 *μ*M BAPTA. The data are expressed relative to total Tau (i.e., Tau-5) levels. (b) Phosphorylated GSK3*β* levels at Ser9 and Tyr216 epitopes were estimated by Western blotting of cell extracts obtained from hippocampal slices treated with 5 *μ*M NMDA for 1 hour. The data are expressed relative to total GSK3*β* levels as percentages of control values and are means ± S.E.M. of 3 measurements per cell extract obtained from 9 different rats. For statistical analysis, one-way ANOVA was followed by Newman-Keuls *post hoc* test. **P < 0.01, ***P < 0.001, drug-treated versus control.

Because NMDA receptor activation is a critical regulator of calcium permeability in neurons [32–35], we investigated whether NMDA-induced decrease of Tau phosphorylation might, in fact, be dependent on calcium mobilization. We observed that preexposure of hippocampal slices to the cell-impermeable form of BAPTA completely blocked the NMDA-induced reduction of Tau phosphorylation at Ser199-202 epitopes (Figure 5(a)), indicating that Tau hypophosphorylation after NMDA receptor activation mainly relies on calcium entrance from the extracellular space. The finding that the effect of NMDA is dependent on calcium entrance in hippocampal neurons predicts that downregulation of Tau phosphorylation at Ser199-202 epitopes could involve inhibition of kinases, such as GSK3*β*, known to influence the phosphorylation of these epitopes [36, 37]. Consequently, the effects of NMDA at the protein level and the phosphorylation status of GSK3*β* were examined. After NMDA treatment of hippocampal slices, total GSK3*β* protein was found to be unchanged, while its phosphorylation status was clearly affected. NMDA not only increased the phosphorylation of Ser9 residues of GSK3*β* but also noticeably reduced phosphorylation at the Tyr216 epitope of this kinase, suggesting functional blockage of GSK3*β* during NMDA treatment (Figure 5(b)).

### 3.3. NMDA-Induced Tau Regulation Relies on PKC Activation

**Figure 6:**
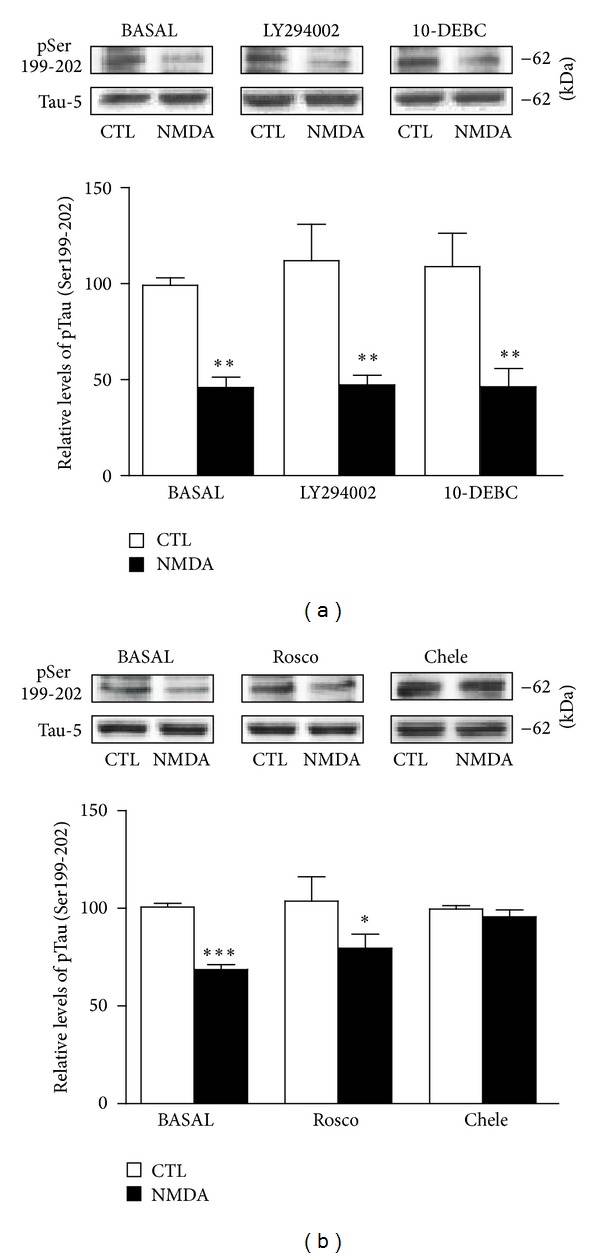
NMDA-induced changes in Tau phosphorylation require PKC activation. (a) Phosphorylated Tau levels at Ser199-202 were estimated by Western blotting of cell extracts obtained from hippocampal slices treated with 10 *μ*M NMDA for 1 hour alone or in combination with the Akt/PKB inhibitor 10-DEBC hydrochloride (10-DEBC; 2.5 *μ*M) and the PI3K inhibitor LY294002 (10 *μ*M). The data are expressed relative to total Tau (i.e., Tau-5) levels. (b) As in (a), except that the cdk5 inhibitor Roscovitine (Rosco; 10 *μ*M) and the global PKC inhibitor Chelerythrine chloride (Chele; 1 *μ*M) were employed. The data are expressed as percentages of control values and are means ± S.E.M. of 3 measurements per cell extract obtained from 6 different rats. For statistical analysis, one-way ANOVA was followed by Newman-Keuls *post hoc* test. *P < 0.05, **P < 0.01, ***P < 0.001, drug-treated versus control.

**Figure 7:**
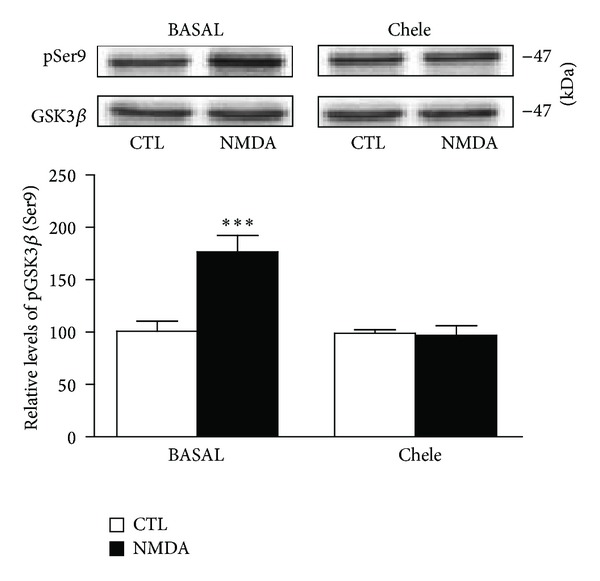
NMDA-induced changes in GSK3*β*  Ser9 phosphorylation are mediated by PKC activation. Phosphorylated GSK3*β* levels at Ser9 and Tyr216 epitopes were estimated by Western blotting of cell extracts obtained from hippocampal slices treated with 5 *μ*M NMDA for 1 hour alone or in combination with the PKC inhibitor Chelerythrine chloride (Chele; 1 *μ*M). The data are expressed relative to total GSK3*β* levels and as percentages of control values. They are the means ± S.E.M. of 3 measurements per cell extract obtained from 7 different rats. For statistical analysis, one-way ANOVA was followed by Newman-Keuls *post hoc* test. ***P < 0.001, drug-treated versus control.

Given that NMDA-induced reduction of Tau phosphorylation seems dependent on GSK3*β* inactivation, we investigated whether NMDA receptors can exert their actions via intracellular pathways known to regulate GSK3*β* through phosphorylation processes [38]. To assess possible roles of PI3K and Akt/PKB pathways in NMDA-induced reduction of Tau phosphorylation, we tested the inhibitor LY294002 (10 *μ*M) and 10-DEBC hydrochloride (2.5 *μ*M), respectively. In these experiments, the inhibitors were applied 30 minutes prior to NMDA exposure to ensure optimal enzymatic inhibition.  Figure 6(a) shows that the ability of NMDA to downregulate Tau phosphorylation at Ser199-202 residues was not affected by these inhibitors. Similarly, we observed that regulation of Tau phosphorylation by NMDA was minimally altered in slices pretreated with the cdk5 inhibitor Roscovitine (10 *μ*M). In contrast, we noticed that the capacity of NMDA to reduce Tau phosphorylation was totally prevented in hippocampal slices preincubated with the PKC inhibitor Chelerythrine chloride (1 *μ*M), indicating that NMDA-induced regulation of Tau phosphorylation probably involves GSK3*β*  blockade by the PKC system (Figure 6(b)). In accordance with this scenario, NMDA-induced enhancement of GSK3*β* Ser9 phosphorylation was not further evident in hippocampal slices preexposed to this PKC inhibitor (Figure 7). Notably, NMDA-induced elevation of GSK3*β* Ser9 phosphorylation was also abolished in slices preexposed to NVP-AAM077 (50 nM), suggesting the contribution of the NR2A subunit (data not shown).

## 4. Discussion

Several lines of evidence indicate that NMDA receptor activation is associated with dephosphorylation of cytoskeletal proteins [9, 11]. For instance, it has been proposed that tonic NMDA receptor stimulation plays a crucial role in limiting MAP2 phosphorylation in hippocampal slices through a mechanism involving stimulation of the calcium/calmodulin-dependent phosphatase, to be PP2B or calcineurin [11, 13]. In this study, we demonstrated that NMDA receptors are also inclined to reduce the phosphorylation levels of Ser199-202 epitopes of 56- and 62-kDa Tau isoforms, an effect which probably implicates calcium entrance in hippocampal neurons through NR2A receptors and subsequent GSK3*β* inactivation.

**Figure 8:**
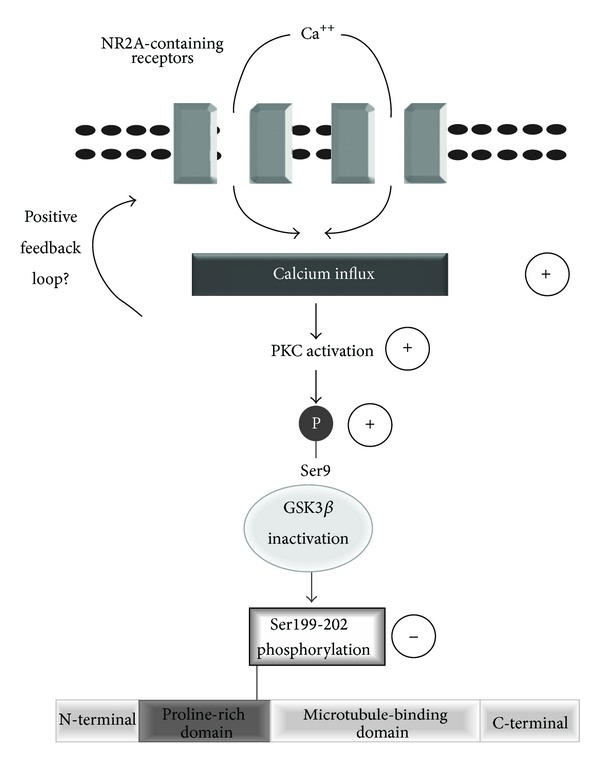
Working model of NMDA-induced reduction of Tau phosphorylation. Activation of NR2A-containing receptors appears to initiate Tau phosphorylation through a mechanism involving calcium accumulation and, consequently, GSK3*β* inactivation via Ser9 phosphorylation by PKC. Through an unknown mechanism, the PKC/GSK3*β* pathway could selectively reduce the phosphorylation of Ser199-202 residues in the proline-rich domain of Tau. Physiologically, activation of NR2A-containing receptors may reduce Tau phosphorylation which could have an impact on protein localisation and aggregation in neurons. The possibility of a positive feedback loop between PKC activation and stimulation of NMDA receptors cannot be excluded [27]. Accordingly, Jones et al. [28] recently demonstrated that activation of the atypical isoform PKC zeta (PKC*ζ*) is selectively coupled with potentiation of NR2A-containing NMDA receptors.

Several signaling pathways, including calcium-dependent ones, have been identified as regulators of the Ser9 phosphorylation state of GSK3*β*, a site which tightly controls both enzymatic activities and other GSK3*β* phosphorylation sites, such as Tyr216 [38, 39]. Along this line, Ortega et al. observed that GSK3*β* activity is downregulated after NMDA treatment of cerebellar granule neurons by mechanisms involving GSK3*β* hyperphosphorylation at the Ser9 residue [40]. According to our results, NMDA-induced reduction of Tau phosphorylation was coupled with decreased GSK3*β* activity, and from a mechanistic perspective, we demonstrated that this effect is possibly dependent on calcium mobilization. The exact mechanisms by which calcium could reduce GSK3*β* activity remain to be clarified, but the existence of pathways whereby calcium-dependent processes can downregulate GSK3*β* activity is plausible. Here, we observed that NR2A-induced Tau dephosphorylation and Ser9 phosphorylation were completely reversed by preincubation of slices in the presence of Chelerythrine chloride, suggesting that the ability of NMDA to reduce Tau phosphorylation likely depends on the activation of classical PKC and subsequent blockade of GSK3*β* activity. Several PKC isoforms are expressed in the hippocampus (for review see [41]), and it remains to be established which enzyme might be more inclined to regulate phosphorylation levels of both Tau and GSK3*β* after NR2A receptor stimulation. A putative biochemical model that accounts for the influence of NR2A receptors and the PKC/GSK3*β* pathway on Tau phosphorylation at Ser199-202 is illustrated in Figure 8. 

Of course, additional pathways that could inactivate GSK3*β* during NMDA receptor activation may exist. Recently, gamma-aminobutyric acid type A (GABA_A_) receptors were implicated in the regulation of Tau phosphorylation at Ser199-202 epitopes. In this particular case, increased Tau phosphorylation at these residues was evoked after GABA_A_ receptor activation by a mechanism requiring cdk5 and, consequently, reduced protein phosphatase 2A (PP2A) association with Tau [42]. However, our pharmacological experiments, described above, suggest that NMDA-induced reduction of Tau phosphorylation did not involve a cdk5/phosphatase pathway as well as other pathways recognized to limit GSK3*β* activity, namely Akt/PKB and PI3K. Among other possibilities, it has recently been demonstrated that GSK3*β* phosphorylation at its Ser389 residue by the highly-enriched enzyme p38 mitogen-activated protein kinase (p38MAPK) attenuates GSK3*β* activity similarly to that implicating Ser9 phosphorylation [43]. Therefore, experiments will be required to precisely establish whether or not NMDA-induced reduction of Tau phosphorylation might be dependent on a pathway involving GSK3*β* inhibition via the p38MAPK mechanism.

Tau proteins contain three (R3) or four (R4) tandem repeats of 31 or 32 amino acids at the carboxyl terminus and 29 or 58 amino acid inserts near the amino terminus [4, 29]. Thus, full-length human cDNA clones have various sequences ranging from 352 to 441 amino acids in length. The adult human brain contains six Tau isoforms, with R3 variants being more abundant than R4 isoforms [17, 44–46]. In rodents, Tau proteins have essentially R4 inserts at their carboxyl terminus with molecular weights ranging from 45 to 65 kDa when run on SDS-PAGE [47]. The present results suggest that not all Tau isoforms are subjected to reduced phosphorylation during NR2A receptor activation, as shown by using an antibody directed against Ser199-202 epitopes. In fact, it is interesting that after NMDA treatments of rat hippocampal slices, reduction of Tau phosphorylation at Ser199-202 sites appears specific for the 56- and 62-kDa isoforms. On the other hand, when compared to other GSK3*β*-targeted epitopes, NMDA treatments did not seem to influence sites located in the proline-rich (Ser 262) and C-terminal (Ser 404) domains of Tau proteins.

The exact molecular mechanisms underlying epitope-and isoform-specific Tau regulation by NR2A-containing NMDA receptors and GSK3*β* are not yet understood. One possible explanation of these selective effects could be the differential localisation of Tau proteins. Interestingly, it was demonstrated previously that the antibody directed against Ser199-202 epitopes (i.e., AT-8) is more inclined to interact with GSK3*β*-targeted Tau proteins located in the somatodendritic compartment of neurons, whereas other antibodies, such as those directed against Ser404 and 396 epitopes, appear to react more strongly with Tau isoforms distributed within axonal pools [46]. In this respect, recent studies have demonstrated that the phosphorylation state of Tau directly impacts cellular localisation [29, 48]. According to Pooler et al. [49], reduced phosphorylation at Ser199-202 epitopes might rapidly favour translocation of Tau proteins to neuronal plasma membranes and, consequently, limit Tau accumulation in the cytoplasmic compartment where it may contribute to neurofibrillary pathology. Consequently, our selective results on Ser199-202 of Tau suggest that the NR2A/PKC/GSK3*β* pathway could mainly interact with Tau proteins located in the somatodendritic compartment. On the other hand, the present studies examining the relationship between Tau phosphorylation and NMDA receptors indicate that NR2A subunits predominantly regulate Ser199-202 epitopes. Of course, the fact that NR2A-induced reduction in Tau phosphorylation was dominant as NMDA concentration increases suggests that NR2A receptors might be strategically located to initiate this downstream signalling. Mechanistically, it has also been reported that high NMDA concentrations can lead to activation of other signaling pathways, such as protein phosphate-1, which has the potential to limit NR2B receptor activation, calcium overload, and neurodegeneration during an excitotoxic event [5]. These possibilities should be further evaluated.

### 4.1. Summary and Conclusions

The current study supports our previous observations that NR2A-containing NMDA receptors are potentially important for controlling Tau phosphorylation in the hippocampus. In fact, the present findings argue that stimulation of these receptors might function as a molecular device limiting Tau phosphorylation in neuropathological conditions, a notion which appears to be consistent with overwhelming evidence that NR2A receptors are coupled to neuroprotective mechanisms likely to reduce cell death [25]. Thus, the fact that NR2A receptor activation is associated with reduction of Tau phosphorylation at Ser199-202 epitopes through GSK3*β* inactivation strongly suggests that the effect reported here may have interesting implications from a therapeutic perspective. Several experiments have demonstrated that hyperphosphorylation is linked with dissociation of Tau from microtubules, accentuating (mis)localisation of Tau proteins in dendrites [48, 50–52]. Our results highlight the need to explore the possibility that NR2A-induced dephosphorylation could limit Tau (mis)localisation in dendrites in response to neuronal insults coupled with, for instance, amyloid-induced neuronal defects [50]. These and other findings might eventually provide critical guidance in the development of better treatments of tauopathies, some of which could be targeted at upregulating NR2A-mediated reduction of GSK3*β* activity by PKC isoforms. Consistent with this scenario is a recent study demonstrating that Tau dysfunction and neurofibrillary tangle formation are abolished by silencing GSK3*β* activity in Alzheimer's disease transgenic mouse models [36], and many other observations indicate that PKC activators can exert antidementic effects during premature ageing of the brain [41, 53–56].

Of course, more detailed studies are required to address whether this NR2A-mediated reduction in GSK3*β* activity could also be important for limiting the progression of other cellular damages (particularly liposomal dysfunctions) which are associated with Alzheimer's disease [57], and further studies are definitely necessary to elucidate whether the NR2A/PKC/GSK3*β* pathway may provide novel perspectives for the treatment of excitotoxicity in pathologies such as ischemic stroke.
